# Attitudinal and demographic factors associated with seeking help and receiving antidepressant medication for symptoms of common mental disorder

**DOI:** 10.1186/s12888-020-02971-9

**Published:** 2020-12-03

**Authors:** Elena A. Manescu, Emily J. Robinson, Claire Henderson

**Affiliations:** 1 Health Service and Population Research Department, King’s College London, Institute of Psychiatry, Psychology and Neuroscience, London, UK; 2 Psychiatry Department, University of Medicine, Pharmacy, Science and Technology, Tirgu-Mures, Romania; 3 Research Data & Statistics Unit, The Royal Marsden NHS Foundation Trust, London, UK

**Keywords:** Help-seeking, Stigma, Attitudes to mental illness, Survey, Common mental disorder

## Abstract

**Background:**

Despite the increased attention given to improvement of mental health-related knowledge and attitudes, rates of help-seeking for mental illness remain low even in countries with well-developed mental health services. This study examines the relationships between attitudes to mental illness, symptoms of common mental disorder and seeking-help and receiving medication for a mental health problem.

**Methods:**

We used data from the nationally representative Health Survey for England 2014 to design three logistic regression models to test for the effects of attitudes to mental illness (measured by a shortened version of the Community Attitudes toward the Mentally Ill, CAMI scale) on: recent contact with a doctor for a mental health problem; use of any type of mental health service in the last 12 months; and having antidepressants currently prescribed, while controlling for symptoms of common mental disorder (measured by the General Health Questionnaire, GHQ). We also tested for an interaction between attitudes to mental illness and symptoms of common mental disorder on the outcomes.

**Results:**

A significant but very small effect of CAMI score was found on ‘antidepressants currently prescribed’ model (OR = 1.01(1.00, 1.02) but not on the two indicators of help-seeking. We also found a significant but very small interaction between CAMI and GHQ scores on recent contact with a doctor (OR = 0.99, 95% CI (0.990, 0.998); adjusted Wald test *P* = 0.01)). Knowing someone with a mental illness had a significant positive effect on help-seeking indicated by: (a) recent contact with a doctor (2.65 (1.01, 6.98)) and (b) currently prescribed antidepressant (2.67 (1.9, 3.75)) after controlling for attitudes to mental illness.

**Conclusions:**

Our results suggest that knowing someone with a mental health problem seems to have a further positive effect on help-seeking, beyond improving attitudes to mental illness. Furthermore, multiple different types and aspects of stigma may contribute to help-seeking behaviours, consequently multi-faceted approaches are likely to be most efficient.

## Background

Although mental illness contributes significantly to the global burden of disease, over two thirds of people affected do not receive any treatment [1, 2]. Only half of people with common mental disorders, i.e. depression (including subthreshold disorders such as dysthymia) or anxiety disorders, will consult their General Practitioner (GP) [3]. Untreated mental disorders are associated with social isolation, higher rates of chronicity, poor quality of life [4] and significant costs [5].

Studies have shown that people who are more inclined to use health services for mental health problems are more likely to be: middle-aged; women; relatively highly educated; not married; with high income; disabled; with more positive attitudes to their mental health care provider; and a greater trust in professional help [6–8].

On the other hand, several factors have been identified to hinder appropriate mental care seeking including two major categories: structural factors, such as inconvenience or inability to obtain an appointment [9]; poor access due to financial barriers [10]; and attitudinal factors defined by many facets of stigma associated with mental illness. A significant proportion of people who experience mental health problems do not seek help, or do so after considerable delay; even in countries with well-developed primary care and mental health services, where structural barriers are low [11, 12]. This suggests that much of this unmet need for treatment can be partially explained by people’s reluctance to seek mental health care [13]. Stigma related to mental illness has frequently been viewed as a key factor in this service use problem [14, 15]. A systematic review conducted in young people indicated stigma as one of the key barriers to help-seeking, together with confidentiality issues, self-reliance and low knowledge about mental health services [16]. People with mental illness have stated that being stigmatized can be worse than the illness itself as its negative effect can continue even after the improvement of their mental state [17, 18]. The concept of stigma is not clearly operationalized. It can be reflected as a dynamic between lack of knowledge, negative attitudes and avoiding or excluding behaviours (discrimination) [19–21]. This multifaceted concept and its relationship to help-seeking, have been evaluated with a variety of instruments [22] but most of the help-seeking outcomes were attitudinal or intentional [15, 23, 24]. Even though attitudes to help-seeking has been reported to correlate with actual service use [25, 26], in practice, however, a considerably lower proportion of those expressing the intention to seek help when dealing with mental health problems actually sought it [27, 28].

A recent review conducted by S. Clement et al. [15], evaluated studies with both attitudinal/intentional and behavioural outcomes on the association between stigma and help-seeking. The majority of attitudinal/intentional studies report a small negative association between stigma and help-seeking. On the other hand, studies that use behavioural measures of help-seeking exhibit much more mixed results, that could partially be explained by ‘reverse causation’ (the probability of the presumed outcome being causally related to the presumed exposure) [15]. The review included also five prospective studies with four of them reporting a negative association but only two with statistical significance.

Studies on clinical populations, regarding the same subgroup of mild to moderate psychiatric conditions, which evaluated perceived public stigma, found it to be correlated with low treatment adherence and early discontinuation [29, 30]. The findings however, were not consistent between age groups; the young reported more perceived stigma but stigma only predicted premature termination of treatment among older patients [30]. Another study evaluated subthemes of stigma i.e. personal and perceived public stigma and reported only personal stigma to be negatively associated with mental health service use [31]. It is not clear yet, how this multifaceted concept of stigma impact on help-seeking.

The limitation of much existing research on actual help seeking and uptake of treatment to clinical populations, who have overcome other barriers to help seeking [14], and the mixed results of studies overall, suggest research at the population level is needed in order to understand how, and to what extent, different facets of stigma related to mental illness may have behavioural impact. This is particularly important as increasing access to care and uptake of treatment when offered are often given as reasons for population based anti-stigma programmes [32] including those focussing on common mental disorder [33].

The aim of this study was to evaluate the relationships between attitudes towards mental illness, as a facet of stigma, and each of help-seeking behaviours and receipt of antidepressant medication, when experiencing common mental disorder symptoms. Our hypotheses were 1) more positive attitudes would be associated with help-seeking and receipt, controlling for symptoms and demographic factors associated with attitudes and/or help seeking, and 2) attitudes will moderate the relationship between symptoms and help seeking, such that among those with more positive attitudes to mental illness, the relationship between symptoms of common mental disorder and indicators of help-seeking and receipt for a mental health problem would be stronger.

## Method

### Data source

Data were obtained from Health Survey for England (HSE) 2014, the 24th cross-sectional survey representative of the general population of all ages living in private households in England. The series of annual surveys have been designed and carried out since 1994 by the Joint Health Surveys Unit of Nicen Social Research and the Research Department of Epidemiology and Public Health at University College London and commissioned by the Health and Social Care Information Centre. The HSE has a core set of questions used every year, plus additional questions to focus on specific topics, which change each year. In 2014 mental illness was one such specific topic, therefore only one wave of the survey could be used. The HSE 2014 dataset was accessed via the UK Data Service.

For survey data collection an interview was conducted, followed by a visit from a specially trained nurse for all those who agreed. The interview was conducted by fully trained personnel using computer-assisted interviewing. Data collected included socio-demographic characteristics, attitudes to mental illness, and use of mental health services. The 12-item General Health Questionnaire (GHQ-12) was also administered as a self-complete measure. A total of 8077 adults aged 16 and over participated in the interview stage of the survey and 5491 of them expressed their consent for a nurse visit. During the nurse visit, participants were asked questions about mental illness experience; familiarity with someone else with a mental illness; and prescribed medicines.

**Fig. 1:**
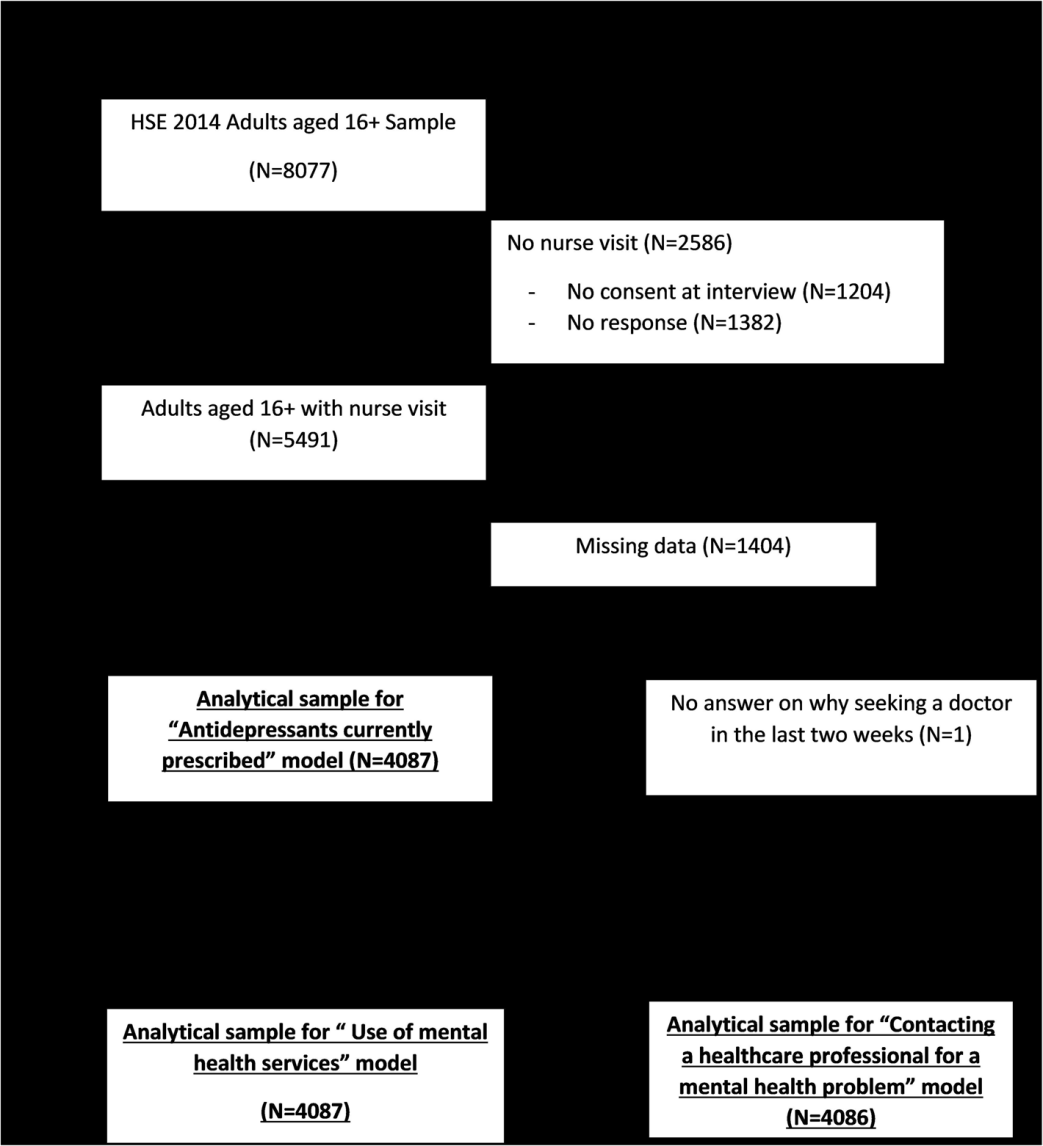
Selection of study sample, Health Survey for England (HSE) 2014

Missing values’ occur for several reasons, including refusal or inability to answer a particular question; refusal to co-operate in an entire section of the survey (such as the nurse visit or a self-completion questionnaire); and cases where the question is not applicable to the participant. Missing values were coded originally in the survey protocol as ‘-9′ and have been omitted from all tables and analyses. Cases of incomplete answers or refusals were excluded from the analysis (Fig. 1).

### Outcome measures

Help-seeking from a professional for a mental health problem was measured using three binary variables derived from a variety of survey questions. Firstly, contact in the last 2 weeks with a doctor for a mental health problem was generated from two questions: participants were asked if they have talked to a doctor during the last 2 weeks, and those who answered yes were then asked a second question about the reason. Possible answers were ‘*physical health problem’*, *‘mental health problem’* and *‘both of these’*. A binary variable was derived by combining ‘*mental health problem’* and *‘both of these’*. Responses of ‘no’ to the first question or ‘*physical health problem’* to the second were combined and used as the reference category. Contact with a doctor included contact with a general practitioner, as well as a specialist or any kind of doctor. Any type of contact i.e. telephone, health centre, home visit, was recorded. The second outcome was use of any type of healthcare service in the past 12 months. Participants were asked about attending a hospital as: an inpatient; outpatient or day patient; through the Emergency department; or any other way, along with the reason for attendance. In addition, a list of mental health community care services was presented and participants were asked if they had used any. Responses to *‘Why were you in hospital?’* of ‘*mental health problem’* or *‘both of these’* (i.e. mental and physical) were combined with any use of mental health community care services. Those who did not attend a hospital or did not use any community care service were combined, together with physical health reasons, and used as the reference. The third outcome variable was having antidepressants currently prescribed, which we interpret as being for common mental disorder, as rates of use for other conditions are much lower [34]. This was derived from a single question and was defined as any versus none.

### Covariates

Attitudes to mental illness were assessed using the 12-item scale derived from the original Community Attitudes toward the Mentally Ill (CAMI) scale developed by Taylor and Dear [35]. The CAMI-12 scale is a subset of the original statements, selected to show levels of mental health-related stigma and tolerance, and first used in the survey evaluating the Time to Change social marketing campaign [36]. This scale was administered in the self-completion questionnaire during the interview visit.

For the HSE 2014, factor analysis was performed on the 12 attitudes statements and generated two internally reliable factors of ‘Tolerance and Support for community care’ and ‘Prejudice and Exclusion’ [37]. The identification of the two factors was consistent with previous research, which ran a factor analysis on the 27 item version of the CAMI questionnaire used in the National Attitudes to Mental Illness survey [38]. A more detailed description can be found elsewhere [39, 40]. The 12 CAMI statements were phrased in both positive and negative directions. Positive views were expressed by agreement with ‘Tolerance and Support’ items, for example ‘The best therapy for many people with mental illness is to be part of a normal community’; and disagreement with ‘Prejudice and Exclusion’ items, such as ‘I would not want to live next door to someone who has been mentally ill’. The degree of a respondent’s agreement or disagreement was rated on a 5-point Likert Scale which was scored as follows: 0 for *‘disagree strongly’*, 25 for ‘*disagree slightly*’, 50 for ‘*neither agree nor disagree*’, 75 for ‘*agree slightly*’ to 100 for ‘*agree strongly’*. Negative statements were scored in reverse so that in each case, a higher score represented a more positive attitude. There was also a sixth option of ‘*Don’t know*. Those choosing this option were excluded from the calculation of the mean score. For most statements, a relatively low proportion of participants gave the response option ‘don’t know’, (between 2-10%), but for one statement, ‘Most women who were once patients in a mental hospital can be trusted as babysitters’, one in five adults chose the ‘don’t know’ response (20%).

A single measure was derived for each of the two factors and takes the average of the mean scores from the individual statements relating to that factor. These variables were derived from valid answers to 6 CAMI items and constitute the two subscales: prejudice/exclusion and tolerance/support [40].

Symptoms of common mental disorder were measured using the General Health Questionnaire 12 item version (GHQ-12) score. The GHQ has been widely used and validated in different settings and different cultures as an effective instrument to screen for common mental disorders [41, 42]. The questionnaire consists of twelve items measuring general levels of happiness, depression and anxiety, sleep disturbance and ability to cope over the last few weeks. Answers of ‘*not at all’* or ‘*no more than usual’* were scored 0 and responses indicating the symptom is present *‘rather more than usual’* or *‘much more than usual’* were scored 1. Consistent with previous HSE surveys, a GHQ-12 score of 4 or more is referred to as a ‘high GHQ-12 score’, indicating probable psychological disturbance or mental ill health [39]. Scores below 4 suggest the absence of mental health problems. Incomplete answers were excluded from the calculation of the GHQ-12 score (Fig. 1).

GHQ-12 score as well as CAMI score were entered as continuous variables to maximise power.

Consistent with previous research [39, 43, 44] the analysis included other factors reported to be associated with attitudes towards mental illness or to help-seeking or receipt. Demographic factors and familiarity with mental illness were included in the regression models as follows: gender, age, educational qualification, income, employment status, ethnicity and familiarity with a mental illness. Age was included as a categorical variable because it has previously been found to show a nonlinear relationship with attitudes to mental illness [45]: *‘16–24’*; *‘25–44’; ‘45–64’* and *‘65+’*. Highest educational qualification was recorded in four categories using the public examinations held in England taken at age 16 and 18, National Vocational and university qualifications: ‘*NVQ4/NVQ5/Degree or equivalent’; ‘Higher education below degree (inclusive A level)’; ‘NVQ2/GCE O Level /NVQ1/CSE other grade equivalent’* and *‘None’*. The measure of equivalised household income was used, which takes into account the number of adults apart from the reference person; and the ages of dependent children in the household as well as overall household income. Based on this measure, households are divided into quintiles (fifths) and used as categories. For ethnic group the HSE uses the 18 2011 UK Census categories, which are then grouped into the categories of White, Black, Asian, Mixed and Other. Employment status was recorded as a binary variable with ‘*in work’*/*‘not in work’* categories.

Information about familiarity with a mental illness was drawn from the question ‘*Who is the person closest to you who has or has had some kind of mental illness’*. The response categories included the participant himself/herself, others (immediate family, partners, friends and acquaintances), and no one if the participant did not know anyone with mental illness. Endorsing the view that one has a mental illness is strongly associated with more positive attitudes [46] and with help seeking and receipt [47], while knowing someone else is associated with more positive attitudes but not as strongly as is personal experience [46].

### Statistical analysis

The analysis followed the HSE design of multistage stratified probability sampling using postcode sectors as the primary sampling units (PSUs) and Postcode Address File as the sampling frame for households. Data were weighted for dwelling unit and household selection and for population profile including age, sex, ethnicity and regional structure of England’s population. A non-response adjustment was added for interview visit as well as for the sample that received the nurse visit. The regression models used to calculate the nurse visit weight include as covariates: age group by sex, household type, region, social class of HRP, smoking status (for adults) and general health. The full sampling design is described in the HSE report for 2014 [37].

Fifteen percent of participants did not provide details of their income and less than 1% had missing information on ethnicity, educational qualification and employment status.

We calculated basic descriptive statistics for participant characteristics in addition to help-seeking variables, CAMI-12 and GHQ-12 scores.

For each of the three outcomes, we ran a logistic regression model with and without the interaction between total GHQ-12 and CAMI-12 scores. Socio-demographic characteristics and familiarity with mental illness were controlled for. Statistical significance of the interaction was tested using the Wald test. Statistical analysis was conducted using Stata V.14.2.

## Results

Table 1 provides details of participants’ sociodemographic characteristics, familiarity with mental illness in addition to General Health Questionnaire (GHQ-12), Community attitudes towards mental illness (CAMI-12) scores and help-seeking variables. Nearly 15% (14.7%) of participants reported symptoms of common mental disorder over the cut point (GHQ ≥ 4) as measured by GHQ-12 and the most frequently reported symptom was ‘*feeling constantly under strain’.*

**Table 1:** Participants’ characteristics, Community Attitudes to Mental Illness (CAMI-12) and General Health Questionnaire (GHQ-12) scores and responses to help-seeking indicators (unweighted frequency and weighted percentage). Analysis sample (*N* = 4087)

Gender n (%)	
Female	2265 (51)
Male	1822 (49)
Age, years: mean (SD)	50.3 (0.2)
Age group n (%)	
16–24	311 (13.6)
25–44	1287 (35.2)
45–64	1448 (32.7)
65+	1041 (18.9)
Ethnicity n (%)	
White	3770 (90.3)
Black	77 (2.1)
Asian	175 (5.4)
Mixed	40 (1.3)
Other	25 (0.8)
Educational qualification n (%)	
NVQ4/NVQ5/Degree or equivalent	1161 (29.8)
Higher education below degree (inclusive A level)	1153 (29.3)
NVQ2/GCE O Level /NVQ1/CSE other grade	996 (24.3)
None	777 (16.5)
Equivalised income n (%)	
Highest Quintile	1874 (21.66)
Second Highest Quintile	1797 (22.76)
Middle Quintile	1660 (21.01)
Second lowest Quintile	1337 (15.71)
Lowest Quintile	1546 (18.86)
Employment status n (%)	
In work	2280 (59)
Not in work	1807 (41)
Familiarity with mental illness n (%)	
Self	183 (3.9)
Other	2675 (66.1)
None	1229 (29.9)
CAMI-12 Score, mean (SD)	74.8 (0.2)
GHQ-12 Score	
median	0
mean (SD)	1.42 (2.6)
Recent contact with a doctor for a mental health problem n (%)	
Yes	66 (1.5)
No	4020 (98.5)
Use of health services for mental health problems n (%)	
Yes	333 (8.1)
No	3754 (91.9)
Antidepressants currently prescribed n (%)	
Yes	438 (9.3)
No	3649 (90.6)

### Factors associated with seeking and receiving help

Table 2 shows results of logistic regression analysis, investigating the relationship between GHQ-12 score, CAMI-12 score and each of: recent contact with a doctor, use of mental health services in the last year and antidepressants currently prescribed. As expected, symptoms of common mental disorder measured by GHQ-12 score were significantly positively associated with help-seeking and receipt in all three models, respectively (OR = 2.25 (1.61, 3.16); 1.08(0.91, 1.28); 1.17 (0.98, 1.39)). Reporting mental illness as a personal experience was positively associated with help-seeking and receipt in all three models as follows: *‘recent contact with a doctor’ (*OR = 7.48 (2.28, 24.54)), ‘*use of mental health services*’ (OR = 2.78 (1.64, 4.71)), and ‘*antidepressants currently prescribed*’ (OR = 10.94 (6.78, 17.65)). Knowing someone with a mental illness was also associated with help-seeking in two of the three models: ‘*recent contact with a doctor’* (OR = 2.65 (1.01, 6.98)) and ‘*antidepressants currently prescribed*’ (OR = 2.67 (1.9, 3.75)).

**Table 2:** Logistic regression analysis of factors associated with help-seeking for a mental health problem as measured by ‘Recent contact with a doctor’, ‘Use of mental health services in the last year’ and ‘Antidepressants currently prescribed’

	Recent contact with a doctor (*n* = 4086)		Use of mental health services (*n* = 4087)		Antidepressants currently prescribed (n = 4087)	
Predictors	OR (95% CI)	*P*-value	OR (95% CI)	*P*-value	OR (95% CI)	*P*-value
GHQ-12 Score	2.25***(1.61, 3.16)	< 0.001	1.08***(0.91, 1.28)	< 0.001	1.17*** (0.98, 1.39)	< 0.001
CAMI-12 Score	1.00 (0.98, 1.02)	0.57	1.00 (0.99, 1.01)	0.42	1.01** (1.00, 1.02)	0.008
Gender						
Female	0.76 (0.39, 1.5)	0.43	1 (0.75, 1.32)	0.97	1.87*** (1.42, 2.45)	< 0.001
Male (ref)	–	–	–	–	–	–
Age						
16–24	0.92 (0.25–3.39)	0.9	0.72 (0.4, 1.28)	0.27	0.09*** (0.03, 0.24)	< 0.001
25–44	1.73 (0.65, 4.64)	0.27	1.24 (0.81, 1.9)	0.31	1.12 (0.75, 1.65)	0.57
45–64	1.21 (047, 3.11)	0.68	0.87 (0.6, 1.26)	0.45	1.27 (0.92, 1.74)	0.14
65+ (ref)	–	–	–	–	–	–
Ethnicity						
Black	3.2*** (8.32, 1.23)	< 0.001	1.18 (0.46, 3.01)	0.69	0.45 (0.14, 1.47)	0.19
Asian	3.91*** (1.22, 1.25)	< 0.001	1.05 (0.42, 2.61)	0.92	0.21** (0.07, 0.58)	0.003
Mixed	7.8*** (2.03, 3)	< 0.001	0.91 (0.2, 3.98)	0.86	0.36 (0.05, 2.39)	0.29
Other	8.56*** (2.46, 2.98)	< 0.001	0.74 (0.14, 3.69)	0.62	3.33*** (1.13, 9.81)	< 0.001
White (ref)	–	–	–		–	
Educational qualification						
NVQ4/NVQ5/Degree/ or equivalent	1.34 (0.4, 4.48)	0.63	1.25 (0.8, 2)	0.33	0.47**(0.3, 0.73)	0.001
Higher ed. below degree (inc. A level)	2.24 (0.87, 5.77)	0.09	1.26 (0.84, 1.87)	0.24	0.53** (0.36, 0.76)	0.001
NVQ2/GCE O Level /NVQ1/CSE other grade	0.89 (0.33, 2.41)	0.82	1.01 (0.66, 1.56)	0.92	0.7 (0.49, 1)	0.05
None (ref)	–	–	–	–	–	–
Employment status						
In work	1.09 (0.43, 2.75)	0.84	0.48*** (0.32, 0.71)	< 0.001	0.58** (0.43, 0.79)	0.001
Not in work (ref)	–	–	–	–	–	–
Equivalised income						
Highest Quintile	0.7 (0.21, 2.26)	0.55	0.87 (0.5, 1.55)	0.66	0.77 (0.l5, 1.17)	0.23
Second Highest Quintile	0.31 (0.08, 1.2)	0.09	0.87 (0.5, 1.5)	0.62	0.91 (0.6, 1.35)	0.64
Middle Quintile	0.46 (0.15, 1.42)	0.18	1.01 (0.6, 1.7)	0.93	0.87 (0.6, 1.27)	0.48 0.17
Second lowest Quintile	1.03 (0.4, 2.65)	0.95	1.07 (0.67, 1.7)	0.75	1.29 (0.9, 1.88)	
Lowest Quintile (ref)	–		–	–	–	–
Familiarity with mental illness						
Self	7.48** (2.28, 24.54)	0.001	2.78*** (1.64, 4.71)	< 0.001	10.94*** (6.78, 17.65)	< 0.001
Other	2.65* (1.01, 6.98)	0.04	0.11 (−0.18, 0.42)	0.44	2.67*** (1.9, 3.75)	< 0.001
None (ref)	–	–	–	–	–	–

*CI* confidence interval, *ref*. reference category, *GHQ-12* General Health Questionnaire 12 items, *CAMI-12* Community attitudes towards mental illness; *P < 0.05, **P < 0.01, ***P < 0.001

Logistic regression analysis included testing separately for both internally reliable factors (dimensions) of the CAMI score: prejudice/exclusion and tolerance/support for community care. However, there was no difference between the results, thus Table 2 displays only the results using the total CAMI score. A small but significant effect of CAMI score was found on ‘antidepressants currently prescribed’ model (OR = 1.01(1.00, 1.02) but not on the two indicators of help-seeking. All ethnic groups compared to white ethnicity, were significantly associated with lower rates of recent contact with a doctor. A negative effect was also observed for Asian ethnicity and other ethnicity than those specified in the questionnaire, compared to white ethnicity in regard to ‘antidepressants currently prescribed’ model. Employment status was a significant factor associated with help-seeking indicated by the ‘use of mental health services in the last year’ and ‘currently prescribed antidepressants’. Those currently working had a predicted lower probability of help-seeking compared to those not working. A lower probability of having antidepressants currently prescribed was found for those with a higher educational qualification and those being aged 16 to 24 compared to those being aged 65 or over.

### Interactions between attitudes and symptoms of common mental disorder on help-seeking

**Fig. 2:**
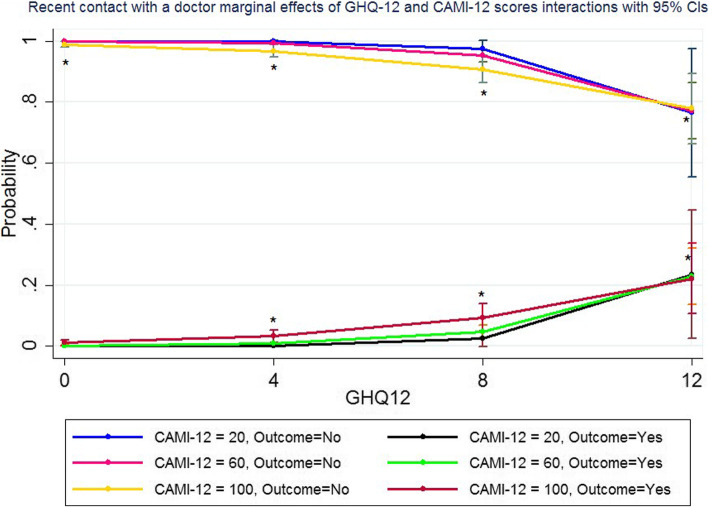
Help-seeking indicated by recent contact with a doctor for a mental health problem marginal effects of General Health Questionnaire (GHQ-12) score and Community attitudes to mental illness (CAMI-12) score interactions with 95% CIs. Legend: *Significant at *P* < 0.05 level

The interaction between attitudes towards mental illness measured by CAMI-12 score and symptoms of common mental disorder measured by GHQ-12 score had a statistically significant but very small effect on help-seeking indicated by the item ‘recent contact with a doctor for a mental health problem’ (OR = 0.99, (0.990, 0.998)); adjusted Wald test *P* < 0.001)). The interaction was not significant for the other two outcomes: ‘use of mental health services in the last year’ and ‘antidepressants currently prescribed’. Fig. 2 shows the predicted marginal effects with 95% CIs of the interaction mentioned above, on help-seeking as measured by ‘recent contact with a doctor for a mental health problem’. Marginal effects shows the changes in the outcome at specified values of CAMI-12 score and GHQ-12 score interaction, while other covariates are held constant.

## Discussion

The results provide mixed support for the hypotheses tested in this study. The statistically significant effect of attitudes towards mental illness on prescribed antidepressants has an associated OR of 1.01, which suggests that this finding may not be clinically relevant. The same is valid for the interaction effect of attitudes found in one of the help-seeking outcomes (‘recent contact with a doctor’).

One interpretation for our results, in the context of the mixed results from other studies reviewed previously [15] is that the influence of stigma may vary depending on either or both of the severity and commonality of the illness. Data from a longitudinal community-based study in Australia showed no correlation between perceived public stigma and actual help-seeking behaviour [48]. The study did not evaluate general stigmatizing attitudes towards mental illness but focused on attitudes towards common mental disorder (depression or anxiety). Consistent with these results, a recent World Mental Health Survey reports attitudinal barriers to be more significant among severe than moderate or mild mental illness [49].

Interestingly, our results showed a statistically significant effect of knowing someone else with a mental health problem on two of the three evaluated categories of help-seeking: ‘*recent contact with a doctor*’ and ‘*antidepressants currently prescribed*’. Consistent with one study in Australian youth [50], these findings suggest that knowing someone else with a mental health problem seems to have a positive impact on seeking care and receiving treatment, independent of any effect of a personal history of a mental health problem or of attitudes towards mental illness. The effect of knowing someone else with a mental health problem may operate via an increase in knowledge about treatment or/and improvement of attitudes towards psychiatric treatment or help-seeking. Together with the lack of association between stigma and help-seeking reported here, the finding of a peer influence suggests that other social influences are stronger on help seeking for symptoms of common mental disorder.

### Strengths and limitations

This study evaluated help-seeking and its associated factors from a retrospective point of view, assessing the actual behaviour, in contrast to most studies evaluating only the intent to seek care or general attitudes about help-seeking [51]. Moreover, the study used a large nationally representative sample and a multidimensional perspective on help-seeking and receipt including three operationalised items for evaluation. Statistically significant results, however, should be considered in the context of a large epidemiological sample, therefore they may not be of clinical significance. Data reported by the present study is limited by the cross-sectional character. Prospective studies are needed to establish a causal direction. Outcomes used in this study were limited to the HSE dataset and may be related to different facets of stigma, for example *having antidepressants currently prescribed* implies not only attitudes towards mental illness as measured by the CAMI scale but also attitudes towards treatment. However, we were only able to evaluate one type which is public stigma expressed by attitudes towards mental illness.

Participants over 65 years old, with lower educational level and income and no familiarity with mental illness have given significantly more ‘don’t know’ responses at two of the CAMI statements (‘Most women who were once patients in a mental hospital can be trusted as babysitters’ and ‘People with mental health problems are far less of a danger than most people suppose’). Nevertheless, we controlled for these characteristics.

Antidepressants are also prescribed for conditions other than mental health such as neuropathic pain and other types of chronic pain, migraine, and fibromyalgia [52]. However, the proportion of those prescriptions while not exactly known assumed to be less than 10% [32]. Compared to prescriptions for mental health conditions when antidepressant prescribing is reported by NHS Digital [53], rates of use for other conditions are much lower thus we interpret *having antidepressants currently prescribed* as being for common mental disorder.

### Implications

Taken together our results suggest that future research consider other factors in addition to stigma when studying help seeking for mental health problems, particularly common mental disorder. For example, a recent qualitative systematic review of attitudinal barriers to help seeking identified the meaning that individuals ascribe to such symptoms as important, i.e. as being related to life problems, along with the value attached to stoicism and mistrust towards health services based on prior negative experiences [54]. Other work has highlighted practical barriers to access due to workforce shortages or cost, including in countries where health systems are well developed [55].

The influence of peer behaviour, i.e. knowing someone else with similar symptoms, appears to be a neglected area in this field. Our findings suggest it is important, while other research suggests it may become increasingly so, at least in England. Rates of familiarity with someone else with personal experience have increased over time [46] in England, reflecting the higher prevalence of common mental disorder in young women [56] and perhaps a greater willingness to disclose personal experience to others [51]. The prevalence of common mental disorder is reported to be increasing further in countries impacted by the covid-19 pandemic. Poorer people already had higher rates of mental ill health [46] and are suffering these impacts more, while Covid-19 presents a relatively greater threat to Black and Minority Ethnic groups and the health and social care workforce; the latter have also been exposed to other severe work-related stresses. People with pre-existing mental illness and those who have had severe illness due to Covid-19 have also experienced worse mental health [57]. Together with our finding that such familiarity is associated with contacting a doctor when dealing with symptoms of common mental disorder as well as the willingness to take antidepressant treatment, these changes in prevalence and familiarity imply that rates of help-seeking will increase, as has been seen for example in counselling services for students [58]. To avoid negative experiences of seeking treatment from under-resourced services, expansion of services is needed to meet this increase in demand.

A further implication for research on help-seeking for mental health problems is the need to distinguish between mild and common problems versus those that are less common and more severe, as the influences on help-seeking for each may differ [49], including the effect of stigma and the influence of peer behaviour. Further, it is important to study whether these influences are changing over time, and whether help seeking for mild and common disorders versus severe mental illness is becoming easier or harder as a result of the overall combination of influences. Population studies from several countries other than England suggests that trends in stigma differ by diagnosis, with that towards schizophrenia worsening while that towards depression remains unchanged [59, 60] In England, the only repeated survey of attitudes measures attitudes to mental illness without specifying diagnoses. While this measure suggests an improvement in attitudes over time, newspaper content analysis suggests that the coverage of schizophrenia has not become less stigmatising in the way that coverage of common mental disorders has [61]. Studies of help-seeking using clinical populations with recent onset of severe disorders are therefore indicated.

## Conclusions

Our results add to the body of evidence, suggesting that for common mental disorders it is important to consider factors in addition to stigma when considering influences on help-seeking. Knowing someone with a mental health problem seems to have a positive effect on help-seeking, beyond improving attitudes to mental illness. Research is needed to understand how and to what extent knowing someone with a mental illness can change help-seeking behaviours, for example whether these are mediated by increased mental health-related knowledge, more positive attitudes towards psychiatric treatment, or a change in perceived social norms regarding help-seeking. This understanding is important, since population level anti-stigma programmes have to decide whether to try and promote familiarity by encouraging discussion among people already familiar with each other, and/or whether to encourage well known role models with personal experience to disclose this in a way that effectively mimics the impact of personal familiarity. Both activities carry potential risks of discrimination for those making the disclosure, and so both safety and how to maximise effectiveness must be considered.

## Data Availability

The HSE 2014 dataset was accessed via the UK Data Service (http://www.ukdataservice.ac.uk). The data are available to students or members of staff at any UK institution of higher or further education (UK HE/FE).

## Abbreviations

HSE: Health Survey for England
CAMI: Community Attitudes towards Mental Illness
GHQ: General Health Questionnaire
